# Meningococcal carriage in UK university students post-pandemic: evidence for MenACWY vaccine impact and ongoing genogroup B risk

**DOI:** 10.3389/fpubh.2026.1917586

**Published:** 2026-08-05

**Authors:** Tyler Harvey-Cowlishaw, Sophie Wartnaby, Jorge Trujillo-Mendoza, Jack L. Clark, Aiswarya Lekshmi, Stephen A. Clark, Simon Royal, Christopher D. Bayliss, David P. J. Turner, Neil J. Oldfield

**Affiliations:** 1 School of Life Sciences, University of Nottingham, Nottingham, United Kingdom; 2 School of Biological and Biomedical Sciences, University of Leicester, Leicester, United Kingdom; 3 Meningococcal Reference Unit, UK Health Security Agency, Manchester, United Kingdom; 4 University of Nottingham Health Service, Nottingham, United Kingdom

**Keywords:** genomic epidemiology, MenB vaccine strain coverage, *Neisseria meningitidis*, oropharyngeal carriage, university students, whole genome sequencing

## Abstract

**Background:**

*Neisseria meningitidis* is a commensal bacterium that asymptomatically colonizes the nasopharynx but can cause invasive meningococcal disease (IMD). Carriage studies are essential to complement disease surveillance and for evaluating the impact of vaccination programs. This study aimed to determine meningococcal carriage prevalence and distribution of lineages in UK university students, a key group at increased risk of carriage and IMD.

**Methods:**

We collected oropharyngeal swab samples from 400 new students at a UK university during Welcome Week (September 2024). Some participants were resampled in November 2024. Culture-amplified polymerase chain reaction (PCR) was applied to detect viable meningococci and determine capsule genogroups. Dot-blot ELISA established isolate capsule expression status (serogroup). Whole genome sequencing established genogroup/clonal complex combinations, population phylogenies, and predicted coverage by licensed group B vaccines.

**Results:**

The September meningococcal carriage rate was 12.5%, which rose significantly to 25.3% in November. The genogroups of recovered isolates were B (34.3%; of which 58.3% expressed capsule), capsule null (24.3%), E (21.4%), Y (12.9%), not genogroupable (5.7%) and W (1.4%). No genogroup C-positive carriers were identified. None of the isolates from genogroups Y or W expressed capsule. For genogroup Y, a statistically significant increase in prevalence was detected between time points (1 to 6.3%). For genogroup B, multiple different clonal complexes were represented, including hyper-invasive lineages responsible for a high proportion of UK IMD cases. The majority of recovered genogroup Y belonged to the β-lactamase-encoding, penicillin-resistant, ST-3587 lineage.

**Conclusion:**

Our study provides information on the meningococcal lineages and serogroups circulating in a key reservoir in the post-pandemic era. The absence of encapsulated genogroup W and Y isolates in this student cohort confirms that the UK’s adolescent MenACWY vaccination program effectively protects young adults from serogroup W and Y carriage acquisition. The presence of genetically diverse genogroup B carriage isolates, including hyperinvasive lineages, supports consideration of strategies to further reduce IMD in at risk student populations.

## 1 Introduction

*Neisseria meningitidis*, an encapsulated Gram-negative diplococcus, is responsible for invasive meningococcal disease (IMD), characterised by rapid onset, severe clinical manifestations, and potential for long-term morbidity and mortality (1). Treatment for IMD usually includes third generation cephalosporins, while prophylaxis is undertaken with rifampicin, ciprofloxacin, or ceftriaxone. Despite the capacity to cause life-threatening illness, *N. meningitidis* is also a common commensal organism of the human nasopharynx with asymptomatic carriage a prerequisite for subsequent transmission and development of IMD. Most worldwide IMD is caused by a limited number of hyperinvasive strains belonging to capsular serogroups A, B, C, W, X and Y (MenA, MenB, MenC, MenW, MenX and MenY, respectively) (2). Infants and children typically experience the highest IMD incidence. A secondary peak is observed in adolescents and young adults who also exhibit the highest carriage rates and hence are considered the critical reservoir and transmitters of meningococci (2, 3).

Polysaccharide-conjugate vaccines targeting serogroups A, C, W, X and Y, and two protein-based vaccines targeting MenB, Bexsero (4CMenB, GSK, Italy) and Trumenba (MenB-Fhbp, Pfizer, USA), have been developed (4). Bexsero includes single recombinant variants of *Neisseria* adhesin A (NadA), neisserial heparin binding antigen (NHBA), factor H binding protein (fHbp) and outer membrane vesicles containing porin A (PorA) (5). In contrast, Trumenba includes two recombinant, lipidated, antigenically divergent fHbp variants (6). Since 2015, the national immunization schedule of the United Kingdom (UK) offers a quadrivalent MenACWY conjugate vaccine to all adolescents aged 13–14 years (individuals remain eligible up to 25 years) and a MenB vaccine, Bexsero, to all infants (7). These vaccine programs have successfully reduced MenBCWY disease incidence by targeting the age groups at risk of serogroup-specific disease or the adolescent reservoirs for transmission depending on vaccine characteristics (8, 9). The schools-based MenACWY immunization program directly protects adolescents against MenACWY disease and additionally provides indirect protection to other age groups (herd protection) due to reduced carriage acquisition (10). The MenB immunization program is an infant-only 2 + 1 schedule of Bexsero. However, studies to date have not detected evidence of reduced MenB carriage following Bexsero immunization and hence the UK program is not expected to generate indirect protection in unvaccinated age groups through reduced transmission (11, 12). Notably, in contrast to polysaccharide-based vaccines, the MenB-targeting vaccines, whilst broadly protective, do not prevent IMD caused by all MenB strains due to the variable presence, sequence diversity, and differing expression levels of the protein antigens across strains (13).

In common with other countries, the incidence of IMD decreased dramatically in England during the COVID-19 pandemic (14, 15). In 2021/2022, removal of measures taken to reduce SARS-CoV-2 transmission led to a sharp upturn in MenB IMD, but not of other serogroups (15). MenB is currently the most common cause of IMD in England across all age groups (16). The initial increase in MenB IMD in 2021/22 occurred mainly among university students and included the “South-West” outbreak, initially associated with students in South-West England. University students are at higher risk of both meningococcal carriage and disease than their non-student peers because of extensive social mixing and residence in high-density accommodation. The post-COVID-19 increase was likely driven by the resumption of social interactions following removal of pandemic restrictions, which increased transmission in a population that may have become more susceptible to MenB carriage acquisition (15, 17).

The relationship between vaccination, natural immunity, social behaviors, and meningococcal carriage remains complex and requires ongoing study, particularly in high-risk populations. University students are a key group at increased risk of carriage and developing IMD. The aims of this study were to examine the prevalence and population structure of meningococci circulating at a UK university in the post-lockdown era and, by examining the characteristics of carried isolates, to provide up-to-date information relevant to meningococcal vaccine programs.

## 2 Materials and methods

### 2.1 Study design and participants

This was a descriptive point prevalence and longitudinal study of meningococcal carriage among first-year home undergraduate students aged 18–25 years at the University of Nottingham, UK. Additional inclusion criteria included residing in a hall of residence during term time and being registered for NHS general practitioner (GP) services at the University of Nottingham Health Centre. Recruitment and initial swabs were collected at events during Welcome Week in September 2024. All 400 participants were invited for follow-up sampling at the NHS health centre in late November 2024. Meningococcal vaccination histories were retrieved from NHS primary care records.

### 2.2 Sampling and laboratory methods

Swabs were collected from the posterior oropharynx by trained medical personnel. Swabs were placed in sterile vials containing 1 mL Amies medium (E-swab, Copan, Italy) and maintained at room temperature. Transport and laboratory processing occurred within 3 h of collection. Sample aliquots (100 μL) were cultured on Lysed GC Selective agar (ThermoFisher, UK) for 48 h at 37 °C in 5% CO_2_. Plate sweeps encompassing all growth (lawns) were stored at −80 °C. Heat-inactivated lawn extracts (boilates) were screened by gel-based multiplex PCR analysis targeting the meningococcal-specific gene *porA* for the identification of *N. meningitidis* and selected capsular locus/genogroup-specific loci (cnl, *csb, csc, csw* and *csy*) to define relevant genogroups, using primers and amplification conditions as previously described (18). Colonies of oxidase-positive, Gram-negative diplococci were subsequently isolated from all *porA*-positive stored sweep samples after growth on Columbia Agar supplemented with Horse Blood (ThermoFisher, UK). A single colony was expanded and subject to re-confirmation of species identification and genogroup by multiplex PCR followed by whole genome sequencing (WGS). Capsular polysaccharide antigen detection (serogrouping) was undertaken by dot-blot ELISA using capsular polysaccharide-specific monoclonal antibodies (19). β-lactamase production was assessed using Intralactam strips (MAST Group, UK) and Cefinase™ β-Lactamase Detection Discs (Becton Dickinson, USA) following manufacturers’ instructions.

### 2.3 Whole genome sequencing and bioinformatic analysis

DNA was extracted using the Wizard Genomic DNA Purification Kit (Promega Corporation, USA). Sequencing (Illumina short read 2 × 250 bp paired end reads) and contig assembly were undertaken by MicrobesNG (https://microbesng.com). Assembled contigs were submitted to the PubMLST Neisseria database (https://pubmlst.org/neisseria/) allowing assignment of alleles, multilocus sequence types (ST) and clonal complexes (cc) (20). Genomes were compared with Genome Comparator using default settings and the *N. meningitidis* cgMLST v3.0 scheme (1,329 loci). Distance matrices were visualized with the SplitsTree App (v6.6.5) using the NeighborNet algorithm. Microreact (v.286) was also used for phylogenomic analysis (using the cgMLST v3.0 scheme) and display of relevant metadata. Predicted coverage provided by MenB vaccines was assessed with the sequence based Meningococcal Deduced Vaccine Antigen Reactivity (MenDeVAR) index (21), which utilizes information from published experimental data (MATS [Bexsero] or MEASURE and/or serum bactericidal data [Trumenba]) to assign vaccine coverage predictions (21). MenDeVAR categorizes predicted protection using the following color scheme: green, isolate harbors ≥1 antigen variant which exactly matches that included in the MenB vaccine (for Bexsero, one of fHbp peptide 1, NHBA 2, NadA 8, or PorA VR2 4; for Trumenba, fHbp peptide 45 or 55); amber, ≥1 antigen is cross-reactive with vaccine-derived antibodies; red, all antigens not cross-reactive to vaccine-derived immune responses; grey, insufficient data available from experimental studies to determine potential cross-reactivity. One genome assembly (isolate 0074H1) was not uploaded to PubMLST due to data exceeding assembly thresholds resulting in sequence-based analyses being restricted to defining MLST/finetyping loci using the PubMLST single sequence query tool.

### 2.4 Statistical analysis

Categorical variables were compared using the chi-squared test (χ^2^) or Fisher’s exact test, as appropriate. Two-tailed *p*-values <0.05 were considered statistically significant. Two-sided 95% confidence intervals (CI) were calculated using Stata v19.5 SE software.

### 2.5 Ethics statement

Ethical approval was obtained from the London - South East Research Ethics Committee (Ref. 23/PR/0741) and written informed consent was obtained from all participants. The study was conducted in accordance with the principles of the Declaration of Helsinki.

## 3 Results

All participants (*n* = 400) were recruited during Welcome Week in September 2024 at the University of Nottingham. Participants were new home undergraduates (i.e., highly likely to have received the MenACWY vaccine through the UK schools program), living in residential halls (known high transmission settings) and registered for GP services at the on-campus health centre (facilitating access to vaccination histories). Participants were screened for meningococcal carriage via oropharyngeal swabbing and invited back for follow up sampling. A set of 79 participants returned for a second swab in November 2024. The overall meningococcal carriage rate at baseline in September 2024 was 12.5% (50/400), which rose significantly to 25.3% (20/79; *p =* 0.0032) in November 2024. Genogroup B was the most common genogroup detected (24/70; 34% of meningococcal-positive samples). Only one genogroup W-positive, and no genogroup C-positive, samples were identified. For some genogroups, statistically significant differences in prevalence were detected between time points, most notably an increase in genogroup Y from 1 to 6.3% (Table 1).

**Table 1:** Characteristics of meningococcal carriage in first-year university students, University of Nottingham, 2024.

Genogroup	Timepoint				*p* value
	September (*n* = 400)		November (*n* = 79)		
	*n*	% (95% CI)	*n*	% (95% CI)	
B	21	5.3 (3.1–7.4)	3	3.8 (0.0–8.0)	0.59
cnl	11	2.8 (1.2–4.4)	6	7.6 (1.8–13.4)	0.03
E	10	2.5 (0.8–4.0)	5	6.3 (1.0–11.7)	0.07
Y	4	1.0 (0.0–2.0)	5	6.3 (1.0–11.7)	<0.01
W	0	0.0 (0.0–0.0)	1	1.3 (0.0–3.7)	0.02
NG	4	1.0 (0.0–2.0)	0	0.0 (0.0–0.0)	0.37

cnl, capsule null locus; NG, not genogroupable.

Of the 79 participants who provided both swabs, 74.7% (59/79) were non-carriers at both time-points, 20.3% (16/79) acquired carriage between the September and November timepoints, while 5.1% (4/79) carried meningococci of the same genogroup at both timepoints. No loss of carriage or genogroup replacement events were detected.

Analysis of vaccination histories showed that 63% (241/382) had a record of MenACWY vaccine administration. As expected, most received the vaccine in the 2019/2020 academic year corresponding to year 9 for the students joining university straight from school (133/241; 55%), with some receiving the vaccine in the preceding (40/241; 17%) or subsequent (44/241; 18%) academic years (Supplementary Figure S1). No records showed administration of a MenB vaccine.

**Figure 1:**
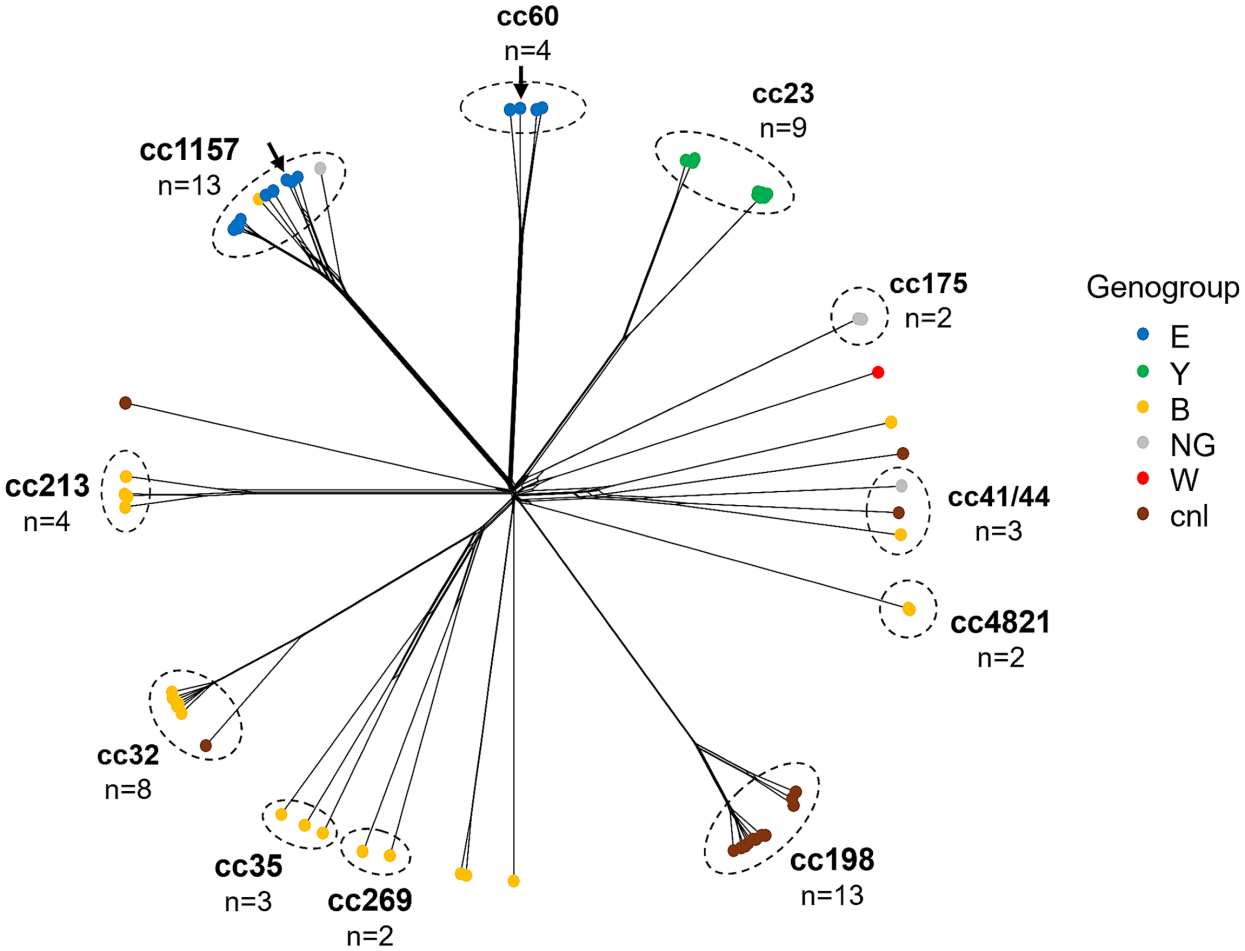
Phylogenetic relationships of meningococcal carriage isolates recovered from first-year university students, University of Nottingham, 2024. Genomes were compared using the *N. meningitidis* cgMLST 3.0 scheme. The resulting distance matrices were assessed with SplitsTree version 6.6.5 using the NeighborNet algorithm to construct the tree. Each dot represents a genome, the color of the dot indicates the capsular genogroup of the isolate. Clonal complexes represented by ≥ 2 isolates are highlighted with a dashed circle with the number of isolates assigned to each CC indicated. The black arrows indicate two isolates assigned to their respective CC’s based on cgMLST analysis. Cnl, capsule null locus; NG, not genogroupable.

As described in Supplementary Table S1, a total of 32 different MLST sequence types (ST), distributed across 14 clonal complexes (cc) were identified from the 70 isolates. Six isolates were not assigned to a cc by the PubMLST database; two of these were assigned based on cgMLST analysis (Figure 1). Overall, 57% (40/70) of isolates belonged to one of four genogroup/cc combinations: cnl:cc198 (13/70; 19% of isolates); E:cc1157 (11/70; 16%); Y:cc23 (9/70; 13%) and B:cc32 (7/70; 10%) (Table 2). The predominant subtypes were P1.18, 25–44 (9/70; 13%) and P1.5–2, 10–2 (6/70; 9%), linked to cnl:cc198 and Y:cc23, respectively. The cnl:cc198 and Y:cc23 combinations were the most common genogroup/cc combination acquired by participants during the study both with rates of 25% (4/16 and 4/16, respectively; Supplementary Table S2). Pairwise comparison of the core genome allelic profiles of paired isolates obtained from participants harbouring meningococci at both sampling points revealed few (≤6) allelic differences confirming longitudinal carriage (Supplementary Table S3).

**Table 2:** Breakdown by clonal complex and genogroup of meningococcal isolates obtained from first-year university students, University of Nottingham, 2024.

Clonal complex	Genogroup (serogroup^a^)						
	B	cnl	E	W	Y	NG	Total
1,157	1 (1)	0	11 (2)	0	0	1	13
198	0	13	0	0	0	0	13
23	0	0	0	0	9 (0)	0	9
32	7 (3)	1	0	0	0	0	8
213	4 (3)	0	0	0	0	0	4
60	0	0	4 (4)	0	0	0	4
Not defined	3 (3)	1	0	0	0	0	4
35	3 (2)	0	0	0	0	0	3
41/44	1 (0)	1	0	0	0	1	3
175	0	0	0	0	0	2	2
269	2 (1)	0	0	0	0	0	2
4,821	2 (0)	0	0	0	0	0	2
11	0	0	0	1 (0)	0	0	1
461	1 (1)	0	0	0	0	0	1
53	0	1	0	0	0	0	1

a Capsular expression detected in vitro for genogroups B, E, W, and Y only.

NG, not genogroupable.

All genogroup Y isolates were from cc23-associated sequence types (ST-3587 or ST-23). Notably, ST-3587 isolates are typically penicillin-resistant due to the presence of a β-lactamase-encoding gene (*bla_ROB-1_*) with some also harbouring a mutated DNA gyrase gene conferring ciprofloxacin resistance (22). Genomic analysis confirmed the six ST-3587 isolates all harbored *bla_ROB-1_* (NEIS3240; allele 2) but wild-type *gyrA* (NEIS1320; allele 1,022) sequences. β-lactamase production by all six Y: ST-3587 isolates was confirmed using both Intralactam strips and Cefinase™ β-lactamase detection discs. The single genogroup W isolate belonged to ST-17638, a rarely isolated ST assigned to cc11. Importantly, none of the genogroup Y isolates or the single genogroup W recovered were serogroupable (Table 2). For genogroup B, eight defined clonal complexes were represented, with 63% (15/24) of isolates belonging to the five major cc’s (cc32, cc213, cc269, cc41/44 and cc461) collectively responsible for the majority of MenB IMD in England (15). Capsule expression was detected in 58% (14/24) of genogroup B isolates (Table 2).

**Figure 2:**
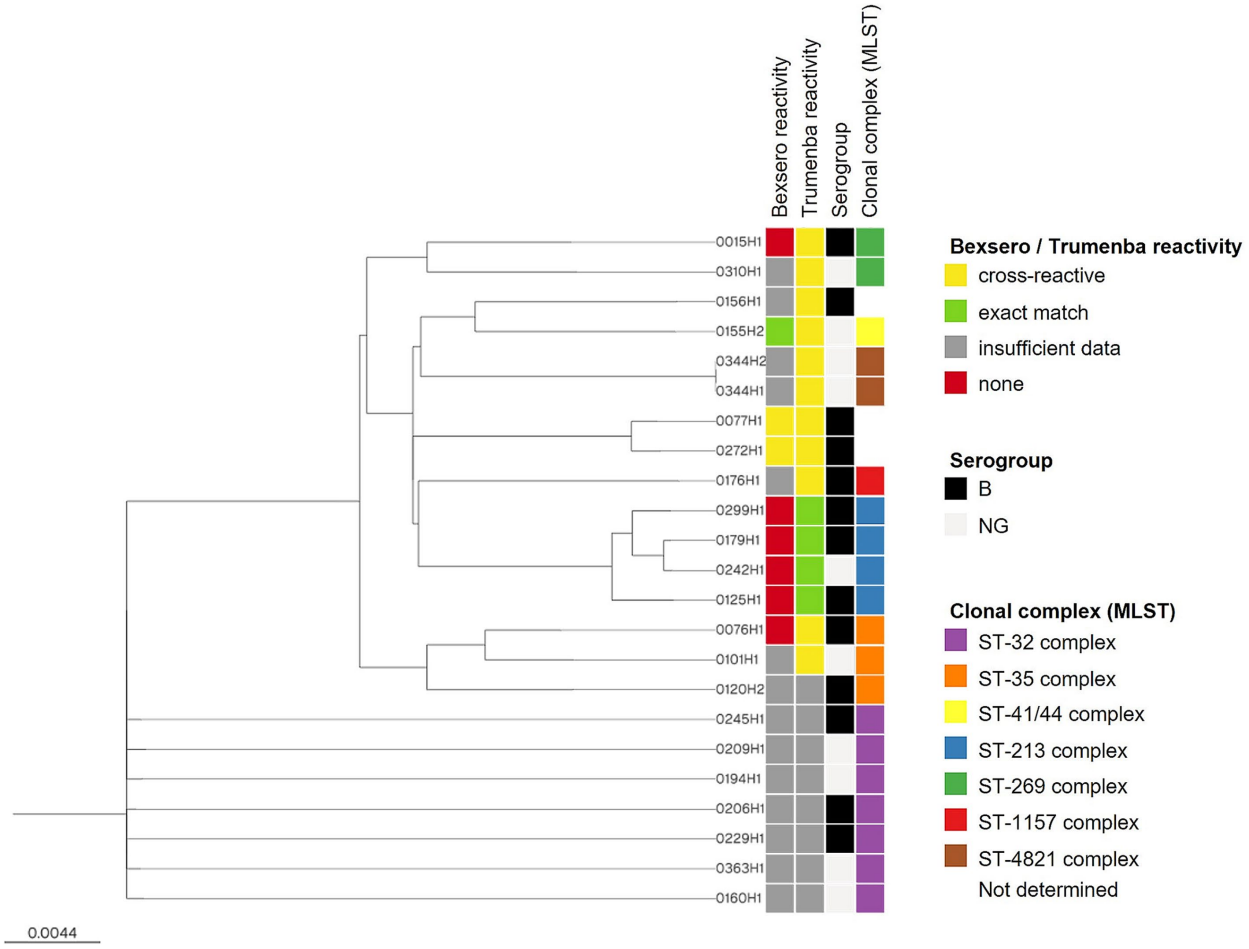
Phylogenetic relationships and MenB vaccine reactivity of meningococcal genogroup B carriage isolates recovered from first-year university students, University of Nottingham, 2024. Phylogeny generated using allele-based core genome MLST (cgMLST) with isolate names at the end of branches. Relevant genotypic and phenotypic data are shown in colored squares. Isolates 0344H1 and 0344H2 (ST-4821 clonal complex) were isolated from the same participant in Sep (H1) and Nov (H2), respectively.

**Figure 3:**
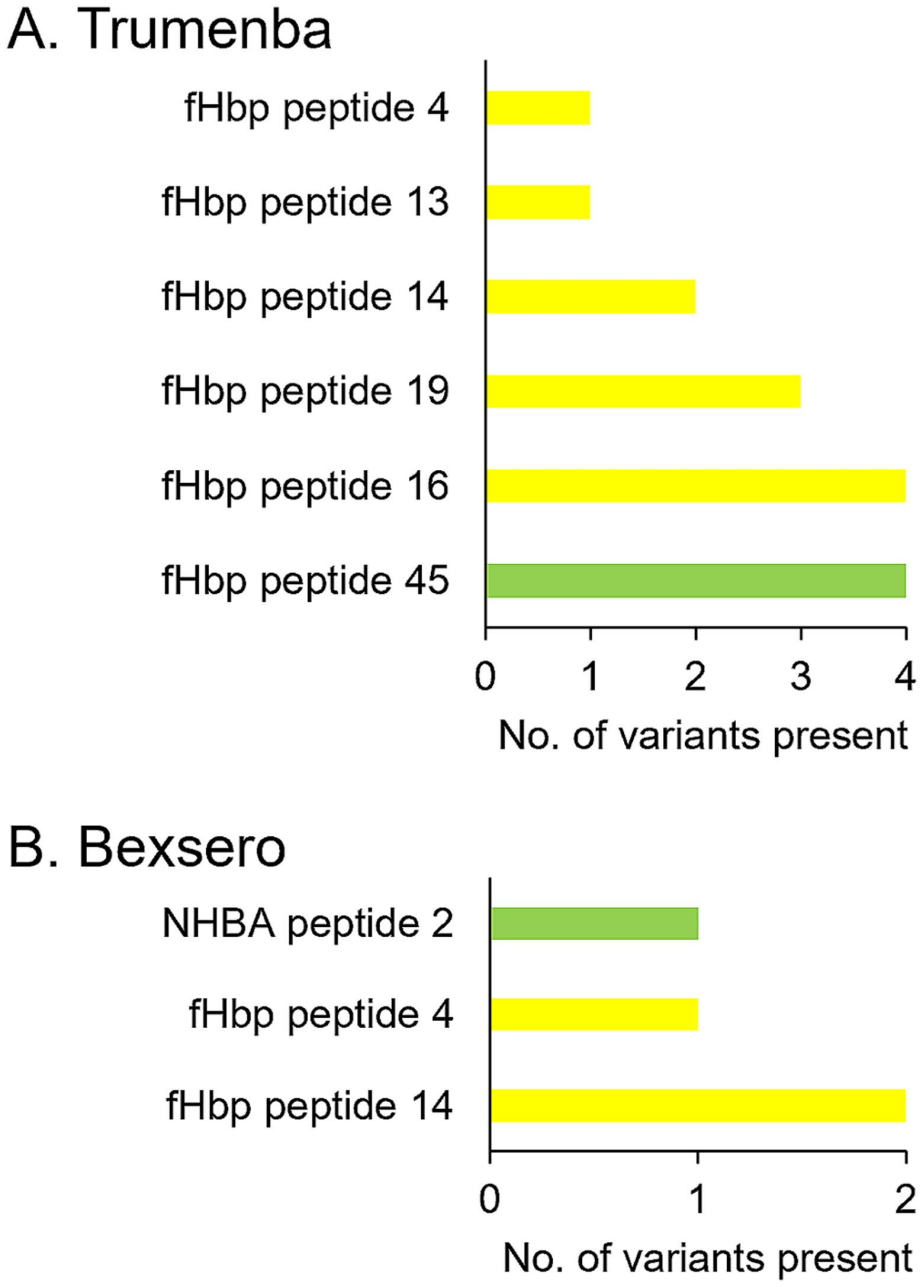
Frequency of matched or cross-reactive MenB vaccine antigen peptide variants present in meningococcal genogroup B carriage isolates recovered from first-year university students, University of Nottingham, 2024. Green indicates an exact peptide match between isolate and vaccine antigen; yellow indicates a potentially cross-reactive peptide as determined using the **(A)** MEASURE or SBA assays (Trumenba) or **(B)** MATS (Bexsero) assays. Trumenba was predicted to protect against 65% (15/23) of the genogroup B carriage isolates; only 13% (3/23) of genogroup B isolates were predicted to be covered by Bexsero. For Bexsero, the number of variants is greater than the number of isolates as one isolate (0155H2) encodes two potentially cross-reactive/exact antigens.

Antigenic studies revealed 17 distinct fHbp peptides and 22 distinct NHBA peptides. NadA peptide sequences were only present in 13% (9/69) of isolates, with all eight cc32 isolates harbouring NadA peptide 1, and the W:cc11 isolate harbouring NadA peptide 6 (Supplementary Table S1). Coverage provided by MenB vaccines against the carriage isolates was assessed using the MenDeVAR index (Supplementary Table S1). The analysis suggested that Trumenba would provide protection (exact or cross-reactive fHbp peptide matches) against 65% (15/23) of the genogroup B carriage isolates with the remainder encoding fHbp peptides with insufficient experimental data for the assessment of coverage (Supplementary Table S1 and Figure 2). In contrast, only 13% (3/23) of genogroup B isolates were predicted to be covered by Bexsero, with 61% (14/23) encoding vaccine antigens with insufficient data for coverage assessment and 26% (6/23) explicitly not covered. Notably, Bexsero was not predicted to provide protection against the B:cc213 isolates, whilst an exact fHbp match (peptide 45) suggested these would be covered by Trumenba (Figure 3). Additionally, eight other isolates predicted to be not covered or insufficient data by Bexsero were predicted to be covered or cross-reactive by Trumenba (Figure 3).

## 4 Discussion

The study aimed to examine the prevalence and population structure of meningococci circulating amongst first-time students at a UK university, and to provide up-to-date information relevant to public health interventions such as MenB and MenACWY immunization programs. To our knowledge, this is the first report of a meningococcal carriage study undertaken in young adults in the UK since the COVID-19 pandemic.

Meningococcal carriage rates vary by setting and age, and, in recent years, have displayed a trend towards lower carriage rates among UK teenagers (10). In line with this, a lower meningococcal carriage rate was detected in the 2024 Welcome Week than in September 2015 (12.5% vs. 14.3%, respectively) for a similar cohort at the same site and using similar methodologies (23). As observed previously (e.g., 23, 35, 36), we detected significant increases in meningococcal carriage rates in these newly arrived students over the next 2–3 months at university. This increase was likely due to high levels of social interactions and communal living as we specifically targeted students residing in catered halls of residence. Most notably, an increased prevalence of genogroup Y was detected. As expected, none of the recovered genogroup Y or W isolates expressed capsule given that MenACWY conjugate immunization is known to suppress MenWY carriage in this age group and that the immunization rate in this cohort was at least 63%. The immunization rate for the cohort as informed by NHS primary care records was lower than expected compared to official GOV. UK data for England (80.8%) which could be due to sample size bias or, alternatively, to missing data. Importantly, the suppressive effect of MenACWY immunization on MenWY carriage appears long-lasting as our examination of vaccine histories revealed that >94% of participants with confirmed MenACWY immunization had received the vaccine ≥3 years prior to September 2024. These findings differ from a study performed in 2015–2016, which observed a rapid increase in carriage of encapsulated MenW:cc11 among first-year university students despite 31% MenACWY vaccine coverage (23, 24). Overall, the lack of groupable MenWY isolates recovered in our study is consistent with other studies indicating that vaccinating individuals aged 13 or 14 (school years 9 or 10) is effectively protecting young adults from serogroup WY carriage acquisition (10).

Genomic analyses indicated that most recovered genogroup Y isolates belonged to the ROB-1 β-lactamase-producing lineage, ST-3587. Since 2013, Y: ST-3587 has been identified as causing IMD in North and Central America (22, 25) and, more recently, IMD in Japan and Spain (26, 27). However, these organisms have rarely been isolated in the UK from IMD cases and were not recovered from two large multicenter carriage studies (UKMenCar4 and Be on the Team) undertaken in UK adolescents prior to the COVID-19 pandemic (10, 28). The two Y: ST-3587 carriers detected in September 2024 were recruited on the same day but resided in different halls of residence. Several acquisition events involving Y: ST-3587 strains were detected at the second timepoint. These findings indicate that this strain is circulating and has high transmissibility within a UK student cohort.

UK Health Security Agency (UKHSA) data show that MenB is now responsible for >80% of cases of IMD in England (16). Our study showed that MenB was the most prevalent meningococcal genogroup recovered from newly arrived students at a typical UK university, with ~60% of these isolates expressing capsule, and many belonging to hyper-invasive lineages. Our data support the notion that, with MenACWY conjugate vaccine-mediated suppression of MenWY disease and carriage continuing, future efforts to protect young adults from meningococcal disease should focus on MenB IMD. Currently the Bexsero vaccine is only offered in the UK to babies (born since late-2015), those with certain underlying health conditions, and individuals at higher risk of getting gonorrhoea (7). No MenB vaccine is routinely offered to other age groups due to cost concerns and the lack of herd protection. However, since 2015, several outbreaks of MenB IMD have occurred amongst students and their contacts at UK universities and further education institutions, notably, the “South-West” (2021–2022) and “South-East” (March 2026) outbreaks, caused by strains belonging to sequence types ST-1161 (cc269) and ST-485 (cc41/44), respectively (29, 30). Neither MenB outbreak strain was recovered in this study, suggestive of low prevalence within the UK carriage reservoir in late 2024.

Sequence-based predictions for MenB vaccine coverage were indeterminate for many MenB isolates. This highlights the requirement for additional phenotypic data to improve genotype–phenotype predictions via the MenDeVAR index. We found that 13% (3/23) of genogroupable MenB strains were coverable by Bexsero, while 26% (6/23) were explicitly not covered. For serogroupable MenB strains these numbers were 15% (2/13) and 38% (5/13), respectively. Most non-protected strains (67%; 4/6) were B:cc213. Other studies have also highlighted the limited theoretical and real-life coverage provided by Bexsero against this cc (31, 32) which suggests potential scope for altering Bexsero’s antigenic formulation to further enhance MenB coverage. An alternative approach would be use of Trumenba (licensed for individuals 10 years and older in the UK (33)) as 65% (15/23) genogroupable or 69% (9/13) serogroupable MenB isolates encoded ‘exact’ or ‘cross-reactive’ Trumenba matches, with the others being indeterminate.

Limitations include that the study participants were from one university, therefore the results may not be generalizable to other UK universities and settings. Secondly, commuters and students living off campus were not included, as the study specifically targeted participants living in halls of residence. Thirdly, the collection of vaccine histories via questionnaire was not undertaken but would have been valuable for comparison to NHS primary care records given the lower than expected MenACWY immunization rate for the cohort as informed by the latter. Fourthly, a considerable number of participants were lost to follow-up at the second time-point impacting on the number of samples obtained at the November time-point. Finally, direct plating with swabs could have increased the chance of detecting meningococcal carriage rather than plating out swab transport media several hours after collection (34).

### 4.1 Conclusion

The detection of a β-lactamase-encoding meningococcal lineage not previously evident within the UK carriage reservoir illustrates how regular sampling of the pharyngeal microbiota of young adults can provide early indications of shifts in population structures and highlights the growing threat of antimicrobial resistance. Our findings support the implementation of regular pharyngeal sampling of young adults, coupled with genomic and phenotypic characterization of isolates, to provide on-going carriage surveillance to complement routine UKHSA IMD assessments. The lack of groupable MenWY isolates recovered supports the assertion that the UK’s schools-based MenACWY vaccination program effectively protects young adults from serogroup W and Y carriage acquisition. This study evidenced that MenB lineages were predominant among carriers in a key ‘at-risk’ group not currently offered the MenB vaccine, but who are potential drivers of transmission in the wider population. This snapshot of the circulating hyper-invasive strains and their antigenic profiles in the post-pandemic era suggests that MenB IMD prevention programs should be widened to include young adults.

## Data Availability

The datasets presented in this study can be found in online repositories. The names of the repository/repositories and accession number(s) can be found in the article/Supplementary material.
