# Supplementary material for: Meningococcal carriage in UK university students post-pandemic: evidence for MenACWY vaccine impact and ongoing genogroup B risk
Source: Front Public Health. 2026 Aug 5;14:1917586. doi: 10.3389/fpubh.2026.1917586 (PMC13486292; doi:10.3389/fpubh.2026.1917586)
Supplement: Supplementary file 1 [file Data_Sheet_1.DOCX]

**Supplementary Figure 1.** Breakdown of the number of participants who received the MenACWY vaccine per academic year, University of Nottingham, 2024.
